# Can HIV-1-Specific ADCC Assist the Clearance of Reactivated Latently Infected Cells?

**DOI:** 10.3389/fimmu.2015.00265

**Published:** 2015-05-27

**Authors:** Wen Shi Lee, Matthew Sidney Parsons, Stephen John Kent, Marit Lichtfuss

**Affiliations:** ^1^Department of Microbiology and Immunology, Peter Doherty Institute for Infection and Immunity, The University of Melbourne, Melbourne, VIC, Australia

**Keywords:** HIV, HIV cure, latency, antibody, antibody-dependent cellular cytotoxicity, NK cell

## Introduction

Combination antiretroviral therapy (cART) has transformed the outcome of human immunodeficiency virus-1 (HIV-1) infection from a severe immunodeficiency syndrome to a chronic clinically manageable disease. However, patients require lifelong therapy and are at risk of developing non-AIDS complications (1, 2). Despite viral suppression in patients on cART, HIV-1 persists via the establishment of a latent reservoir. The adverse aspects and burden of lifelong cART together with the establishment of HIV-1 latency substantiate the need for an HIV-1 cure. A cure will either require complete eradication of the latent HIV-1 reservoir (termed “sterilizing cure”), or a sufficient reduction of HIV-1 levels wherein long-term viral suppression can be achieved without cART (termed “functional cure”). A key barrier to an HIV-1 cure is the persistence of latent replication-competent HIV-1 within long-lived resting CD4^+^ T cells (3). Without active HIV-1 replication or antigen expression, these latently infected cells are hidden from cART and are not eliminated by immune responses.

## HIV-1 Cure Strategies

The Berlin patient is widely regarded as a proof-of-concept for an HIV-1 cure (4–6). The patient received a bone marrow transplant from a donor with the homozygous *CCR5* Δ32 mutation, which confers high resistance to most strains of HIV-1 (7). Remarkably, viremia has remained undetectable for 6 years in the absence of cART. Two additional patients (the “Boston patients”), who received transplants from non-HIV-1-resistant donors, only had non-sustained remission periods (3–8 months) where HIV-1 viremia remained absent after cessation of cART before HIV-1 rebounded to high levels (8). Until very recently, early and intense cART intervention during acute HIV-1 infection was postulated to be a feasible cure approach. The “Mississippi baby” was thought cured of HIV-1 after receiving intense cART during acute HIV-1 infection (9). However, HIV-1 rebounded in the baby after 27 months of viral remission in the absence of cART, implying that HIV-1 latency was established early after infection and that latent cells can remain dormant for long periods prior to becoming reactivated (10, 11).

An alternative method for eliminating the latent reservoir is the “shock and kill” approach where latently infected cells are reactivated to express the HIV-1 genome (“shock”) and are subsequently killed by the immune system or viral cytopathic effects (“kill”) (12). cART administered alongside virus reactivation by latency-reversing agents (LRAs) should impede the infection of new cells. Clinical studies are now beginning to understand how to disrupt the HIV-1 latent reservoir. Several histone deacetylase inhibitors (HDACi) have been studied *in vitro* with promising results (13). However, recently completed clinical trials examining the HDACi vorinostat, panobinostat, and romidepsin as LRAs showed only partial success (14–16). Although these HDACi induced a significant and sustained increase in HIV-1 transcription (mRNA) and/or plasma viremia from latency in the majority of HIV-1-infected patients, they failed to decrease total integrated HIV-1 DNA – an indication that the viral reservoir did not change. A more promising study showed that administration of a toll-like receptor 7 agonist as an LRA to simian immunodeficiency virus (SIV)-infected rhesus macaques treated with cART induced transient plasma viremia and resulted in a decrease in total SIV DNA levels (17).

Despite the large research effort investigating approaches to reactivate HIV-1 expression in latently infected cells, there is limited knowledge on the fate of these cells following reactivation. Shan et al. demonstrated that latently infected cells derived from HIV-1-infected subjects that were reactivated with the HDACi vorinostat *ex vivo* did not die from viral cytopathic effects and were not killed by autologous cytotoxic T lymphocytes (CTL), which may have been relatively quiescent in the presence of cART (18). The reactivated latently infected cells were, however, partially killed by autologous CTLs that were pre-stimulated with HIV-1 antigens. Consequently, there is a risk that the surviving reactivated cells may revert back to latency and replenish the latent reservoir. As such, HIV-1 reactivation from latency alone is not sufficient to eliminate the latent reservoir. This suggests that further immune modalities may need to be harnessed to purge latently infected cells. While pre-stimulation of CTLs *ex vivo* could lead to elimination of reactivated latently infected cells, protective CTL responses tend to be restricted by rather uncommon HLA-I alleles (HLA-B27, HLA-B57) (19, 20). Also, a recent study demonstrated that the majority of viruses in the latent reservoir carry CTL escape mutations that render reactivated cells partially resistant to elimination by immunodominant CTL responses (21). Still, appropriate boosting of these CTL responses will most likely be required for the elimination of the latent reservoir, which is difficult with current HIV-1 therapeutic vaccine strategies that have shown only modest success *in vivo* (22–24). Although there may be vaccine strategies [such as cytomegalovirus vector vaccines (25)] that can induce CTLs to non-escaped, unusual and diverse epitopes, this may prove difficult. The efficacious potential of non-CTL immune responses capable of eliminating the latent reservoir needs to be explored.

## Antibody-Dependent Cellular Cytotoxicity Against HIV-1

We postulate that HIV-1-specific antibodies might be able to mediate killing of reactivated latently infected cells through antibody-dependent cellular cytotoxicity (ADCC). HIV-1-specific ADCC involves the binding of antibodies to HIV-1 antigens expressed on the infected cell surface and the subsequent recruitment of innate effector cells, such as natural killer (NK) cells or monocytes (26). The cross-linking of Fcγ receptors on these innate immune cells by the Fc region of IgG antibodies results in the cytolysis of infected cells as well as the release of cytokines and chemokines by the innate effector cells (26–28). Numerous studies have suggested a protective role for ADCC against HIV-1 infection. High levels of HIV-1-specific ADCC antibodies have been correlated with slower disease progression (29–31), and have been shown to play a role in controlling HIV-1 infection in elite controllers, a rare group of individuals able to suppress viremia below detection limits without cART (32). Furthermore, ADCC antibodies have been implicated as an immune correlate in the moderately successful HIV-1 RV144 vaccine trial (33, 34).

## Potential Barriers for ADCC-Mediated Elimination of the Reactivated Latent HIV-1 Reservoir

Although theoretically attractive, whether reactivated latently infected cells can serve as targets for ADCC remains unclear. A major determinant for ADCC responses against HIV-1-infected cells is the availability of cell surface HIV-1 envelope (Env) protein for the binding of ADCC antibodies. Even though results from recent clinical trials of LRAs seem promising, it is not known whether reactivated latently infected cells express sufficient Env antigens on the surface for efficient binding of ADCC antibodies. While the level of Env expressed on the surface is an important factor for anti-HIV-1 ADCC, the conformation of Env and consequent availability of epitopes for the binding of ADCC antibodies might be even more critical. As reviewed in Kramski et al. (35), several recent studies have shown that HIV-1-infected cells can evade ADCC *in vitro* through the actions of the accessory proteins, Vpu and Nef (36–38). Vpu and Nef can downregulate surface CD4 expression, thereby preventing CD4–Env interactions on the surface of infected cells. This leads to the concealment of many ADCC antibody epitopes that are only exposed on CD4-bound Env trimers, thus reducing the level of ADCC antibodies binding to infected cells (38). Furthermore, a recent study reported that the majority of anti-HIV-1 ADCC antibodies in sera from HIV-1-infected individuals recognize CD4-induced epitopes on Env, which are mostly concealed on HIV-1-infected cells (39). In addition, Vpu inhibits the host restriction factor tetherin, which tethers nascent virions to the surface of infected cells. By doing so, Vpu ensures the efficient release of virions and also reduces the level of HIV-1 antigens available for ADCC antibody binding (36, 37). Overall, sufficient HIV-1 antigen expression and epitope availability on reactivated cells through the use of potent LRAs, and perhaps, the therapeutic inhibition of Vpu and Nef will be needed to enhance ADCC antibody-binding to reactivated cells (40).

Another potential barrier for ADCC-mediated elimination of the latent HIV-1 reservoir is that levels of HIV-1-specific ADCC antibodies decline in HIV-1-infected individuals on long-term cART (41). Similar to the waning of HIV-1-specific CTL responses in patients on cART (42), the decline in HIV-1-specific ADCC antibodies likely results from a lack of antigen stimulation as a result of cART-mediated viral suppression. However, the decline in ADCC antibodies is much less substantial than the decline in CTL responses on cART. If ADCC antibodies are indeed capable of eliminating reactivated latently infected cells, it is not known whether the level of ADCC antibodies will need to be boosted prior to latency reversal. Although antibody-inducing protein-based HIV-1 vaccines may be more successful than CTL-based vaccines, their efficacy in the context of HIV-1 cure strategies is unknown. Therapeutic vaccines administered to HIV-1-infected individuals may also only expand pre-existing HIV-1-specific B cells, which could have already induced viral escape from ADCC in the earlier stages of infection (43), similar to that seen for CTLs (21). Further activation of pre-existing B cell immunity toward HIV-1 may also induce undesirable isotype switching, resulting in a switch from ADCC-mediating isotypes (IgG) to non-ADCC-competent isotypes (IgA) (34, 44). Thus, instead of attempting to stimulate immune responses in HIV-1-infected individuals who may have partially compromised immune systems, the passive transfer of ADCC-mediating monoclonal antibodies might be a more promising and easier approach.

Over the years, a series of potent and broadly neutralizing Env-specific monoclonal antibodies (bNabs) have been isolated from HIV-1-infected individuals (45–47). Many of these bNabs have been shown to mediate ADCC as well. Barouch et al. and Shingai et al. showed that the passive transfer of certain bNabs, either individually or in combination, was able to induce suppression of viremia in macaques chronically infected with chimeric simian-HIV (SHIV) (48, 49). Although not tested directly, the demonstration by Barouch et al. (48) that these bNabs could result in a reduction in proviral DNA suggests that ADCC against SHIV-infected cells may have been important in reducing viremia. Halper-Stromberg et al. demonstrated that administration of a combination of bNabs followed by a combination of LRAs could result in decreased viral rebound from HIV-1 latently infected cells in a humanized mouse model of HIV-1 infection (50). These results suggest that the passive transfer of ADCC-competent antibodies to patients, along with LRAs, could result in ADCC-mediated elimination of reactivated latently infected cells and purge the latent reservoir. Similar to the use of multiple drugs for cART, administration of a combination of multiple monoclonal antibodies is most likely to prevent emergence of viral escape mutants. Since Env is likely to be in the native closed trimeric conformation due to CD4-downregulation by Vpu and Nef, and HIV-1-infected individuals predominantly have serum ADCC antibodies that recognize CD4-induced epitopes on Env (39), ADCC-competent monoclonal antibodies that bind to non-CD4-dependent epitopes on Env are highly desirable. Future studies need to assess whether (1) ADCC is a potential immune mechanism to eliminate reactivated latently infected cells *in vivo*, and (2) if native Env trimer-binding, ADCC-competent bNabs can eliminate latently infected cells following administration of LRAs.

Even if ADCC antibodies can efficiently recognize HIV-1-infected cells, the demonstration that autologous CTLs from patients on cART were unable to kill reactivated latently infected cells without prior antigen stimulation raises concerns that NK cells in these patients may not be able to eliminate the reactivated reservoir either (18). While some studies have shown that NK cells in HIV-1-infected patients remain capable of mediating ADCC (51, 52), other studies demonstrate that the NK cells may become “exhausted” and exhibit reduced functionality due to chronic immune activation (53, 54). Exhausted NK cells may express lower levels of CD16 (55), reduced levels of NK cell activating receptors (53), and have impaired CD16 signaling (54). Indeed, we have demonstrated that CD16-downregulation is induced by activation of NK cells in an anti-HIV-1 antibody-dependent manner (56, 57). This downregulation is mediated by matrix metalloproteinases (MMPs) and inhibition of MMPs can prevent CD16-downregulation or restore CD16 expression on previously activated NK cells (55, 56) Thus, immune modulating strategies including MMP inhibition, *in vivo* cytokine therapy, or adoptive NK cell therapy may need to be implemented to boost NK cell functionality before latency reversal to unleash the full potential of ADCC antibodies in cure strategies.

## Conclusion

Despite the increasing efforts funneled into HIV-1 cure research, an HIV-1 cure still remains elusive. The relapse of the “Boston patients” and the “Mississippi baby” after months of viral suppression emphasizes the difficulty of the task ahead. Recent studies show the potential utility of CTL immune responses in clearing reactivated latently infected cells. Moving forward, CTL functionality in cART-treated patients may need to be boosted and directed toward sub-dominant epitopes. In addition to CTL responses, ADCC represents another immune mechanism and further investigation to assess its potential to clear reactivated latently infected cells is warranted. Advances in HIV-1 cure strategies will require an improved understanding of not only how to reactivate HIV-1 from latently infected cells, but also how to clear them with the most effective immune responses.

## Conflict of Interest Statement

The authors declare that the research was conducted in the absence of any commercial or financial relationships that could be construed as a potential conflict of interest.
